# Can impersonal touch replace interpersonal touch? An investigation using the rubber hand illusion

**DOI:** 10.1371/journal.pone.0319433

**Published:** 2025-05-07

**Authors:** Jaehyoung Bae, Christian Wallraven

**Affiliations:** 1 Department of Brain and Cognitive Engineering, Korea University, Seoul, South Korea; 2 Department of Artificial Intelligence, Korea University, Seoul, South Korea; Universitat de les Illes Balears, SPAIN

## Abstract

Our socio-emotional development and well-being critically depends on interpersonal tactile interactions, which are sensed by the skin through C-tactile (CT) afferents that respond to gentle, slow touch at typical skin temperatures. In the present study, we investigated whether impersonal touch would be able to provide similar pleasantness compared to interpersonal touch within a body-ownership illusion paradigm. To provide impersonal touch at similar parameters, we used a thermal probe kept at $32^{\circ }$C (typical skin-to-skin temperature) compared to a flat hand as interpersonal touch. Both forms of touch were performed at CT-compatible speeds of 3cm/s by a male trained experimenter within a classic rubber hand illusion (RHI) paradigm in two counter-balanced within-participant conditions. A sample of N=45 healthy participants was tested and pleasantness ratings, touch deprivation, and the Need-For-Touch-Scale (NFT) were gathered. Overall, the illusion was similar in both touch conditions and, importantly, we found no statistically significant difference in pleasantness between interpersonal and impersonal touch. Interestingly, neither NFT scores, nor touch deprivation measures correlated with individual differences in the RHI and affective ratings. Our results suggest that impersonal touch with CT-optimal components provide a pleasantness and subjective illusion experience compared to interpersonal touch under the RHI paradigm.

## Introduction

Touch is the primary way to interact with the environment as well as other individuals. Research into tactile processing, however, has often focused on studying its discriminative aspects, viewing the skin as an exteroceptive organ that mainly detects, discriminates, and identifies stimuli [1]. Apart from its discriminative function, however, the sense of touch also serves important interoceptive and affective functions that form the basis for interacting with other people through interpersonal or social touch [2–4], for example. Unlike other social signals including facial expressions, touch may occur less frequently, nevertheless the effect of interpersonal touch is used to show affection and support and as such can fundamentally influence social interactions [5]. If the context between touch giver and receiver is matched, interpersonal touch is regarded as a powerful affective signal [3, 6]—this type of touch is an innate behavior that promotes relaxation by decreasing stress-related sympathetic activation, thereby satisfying a fundamental need in the regulation of physical and emotional well-being [7, 8].

Affective touch—as the main topic of the present work—is a form of gentle interpersonal touch able to buffer stress and to be both calming and relaxing [9, 10], inducing a hedonic effect as its main emotional component [9]. Previous studies indicated that affective touch may be mediated by specialized sensory pathways: slow, low-force stroking movements at typical skin temperatures were found to activate the so-called C-tactile fiber systems (CT [11, 12]) whose afferents are located mainly in hairy [13, 14] and to a lesser extent also in glabrous skin [15]. To optimize these affective responses, affective touch should be performed as a stroking movement on hairy skin with a gentle, slow speed [16, 17]: specifically, stroking velocities of 1–10  cm/s with skin-to-skin contact temperature of $32^{\circ }$C on hairy skin have been shown to yield maximum responses from the CT afferents in tandem with strong affective ratings [9, 12, 18–20].

Further studies have tried to characterize the processing and attribution of hedonic qualities to affective touch. Although the CT afferents pathway is considered an innate processing route [4], affective touch can be modulated by top-down processing [21], such as attribution of touch to a male or female, for example [22]. Similarly, interpersonal touch stimulation with a happy face together is regarded as the most pleasant touch compared to an angry or neutral face [23]. In addition, the same gentle stroke of the skin can provoke various reactions depending on surrounding affective cues, the situation, the touch source [24], prior touch experience or cultural background [25]. At the same time, similar response patterns were found for pleasantness ratings of observed stroking to a human arm with an actual human hand, mannequin arm, robot arm, and even a plastic object [26].

The question of whether affective touch may even be influenced by body-ownership has also been addressed by a few recent studies. One of the most widely used experimental paradigms for modulating body-ownership in this context is the so-called rubber hand illusion (RHI, [27]): in classic versions of the RHI, participants may experience body-ownership of a fake part of the body (rubber hand) when seeing it being stroked while experiencing synchronous stroking at the corresponding area on their own body hidden from sight. The sense of body-ownership relies on both exteroceptive (external) and interoceptive (internal) information [28]. Affective touch, which stimulates CT afferents, sends interoceptive signals to the brain, specifically the insula [19, 29], a region involved in body-ownership and sensory integration [30]. This connection suggests the need to investigate affective touch to understand its role in body-ownership perception. Some studies have reported correlations between affective touch and body-ownership illusions [28, 31, 32] and that affective touch is able to even enhance the effect of the illusion (e.g., [28, 33]; but see [34, 35]), however, the mechanisms behind the effectiveness of affective touch in modulating the intensity of RHI need further study.

The high degree of context-dependency of affective touch would suggest that the interpretation of the touch environment may be more dependent on the touch receiver than on the stimulus that applies the touch. As a result, it may be possible to induce affective touch by proper stimulation through non-human (impersonal) means by applying force, material, or warmth. Experiments with rotating brush stimulation devices have shown that increased CT afferent activation went along with increased perceived pleasantness of the touch stimulus (e.g., [36, 37]). Additional actuators that can stimulate receptors for affective touch employ methods such as rotational friction, air puffs, vibration, or thermal-probe stimulation [20, 38]. The degree to which such impersonal touch may be able to fully replace the interpersonal touch, however, remains unclear.

The primary aim of the present study was to investigate how pleasantness differs between interpersonal and impersonal touch under similar conditions. For this, we made use of the rubberhand-illusion paradigm in which we applied touch via a custom-designed thermal probe, comparing this to human touch in terms of perceived pleasantness under the RHI body-ownership modulation. Since visual stimuli are considered to dominate the overall affective qualities of multi-modal stimuli [39], the available range of pleasantness evoked by these combined sensory inputs can be enriched by combining multiple factors of the affective touch like touch velocity and thermal input with the same visual touch on the rubberhand (e.g., [40]). The main metric used to describe the differences of pleasantness and the RHI effect between the two touch conditions was subjective self-report via a 7-point Likert scale. This allows participants to rate their experience of affective touch under the RHI body-ownership modulation. We expected that providing impersonal touch at similar parameters would induce pleasantness and enhance the illusion to levels comparable to interpersonal touch, yielding the null and alternative hypotheses of:

- **H0:** There is no difference in pleasantness between interpersonal and impersonal touch under similar conditions.
- **H1:** Interpersonal touch will yield different pleasantness compared to impersonal touch.

## Materials and methods

### Participants

The sample size was determined using a priori power analysis using G*power (G*Power 3.1, [41]). Previous publications [42, 43] comparing pleasantness between interpersonal and impersonal touch reported effects sizes from 0.80 to 1.15, from which we determined an average effect size of 0.98. Using a within-participant paired t-test, and a desired power at 0.80 with a Type-I error rate threshold of 0.05, this led to a sample size of *N* = 11 participants. Additionally, we planned a further investigation of the relation between touch deprivation and perceived pleasantness using linear regression. For this, another a priori sample size calculation was conducted based on a previous study [44]. Considering the reported effect size of 0.344, and a desired power at 0.80 with a Type-I error rate threshold of 0.05, the minimum required sample size for the linear regression analysis was determined to be *N* = 25 participants. To ensure sufficient statistical power for both analyses, a combined sample size of a minimum of *N* = 36 participants was considered for the study. To cope with potential dropout and account for outlier case exclusion, we recruited an additional 20% of participants.

A sample of forty-five healthy participants (23 women; mean age = 23.8 ± 2.5, range 19–30) were thus recruited from Korea University for the study. Participants were right-handed, had normal or corrected-vision, native Korean speakers. In addition, they were all naïve with respect to the RHI. Informed, written consent was obtained from all participants before the experiment, which was approved by the Korea University Institutional Review Board (KUIRB-2020-0254-01), and was performed in accordance with the Declaration of Helsinki.

### Procedure

**Fig 1:**
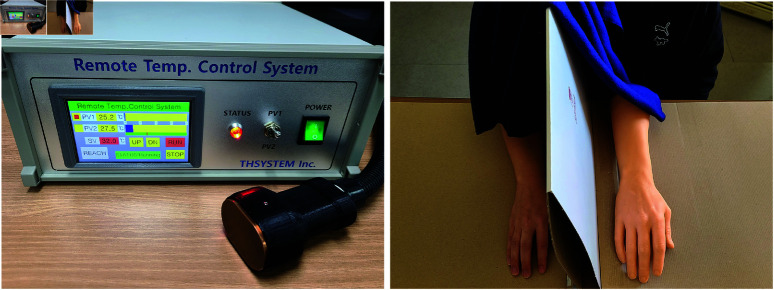
Experimental setup. (a) The thermal probe. (b) The real and fake hands.

In the experiment, the “classic” version of the RHI paradigm designed by Botvinick and Cohen [27] was adopted. The participants were unaware of the experiment’s purpose. They were seated comfortably on a chair and were adapted to the experiment room temperature of $28^{\circ }$C before starting the experiment procedure. Subsequently they were asked to place and fix their right forearm on the right side of a divider in front of them with the palm facing down (see Fig 1b). A rubber hand was placed on the left side of the divider, such that the distance between the participant’s right index finger and the index finger of the rubber hand was 25 cm horizontally. To fully hide the participant’s right forearm from sight, a blanket was placed on their right shoulder. During the experiment setup time, the experimenter conducted a short conversation with the participants to avoid the context of a “stranger touching me” [45, 46]. Prior to initiating the RHI stimulation, participants were informed of the experimental procedure, including the area where stroking would be applied—but not whether this would be done with a thermal probe or via the experimenter’s hand, nor about the potential for RHI to create expectations in their responses (i.e., “A participant should expect the illusion for synchronous induction.” [47]).

Participants underwent two conditions in a counterbalanced within-subject design, the interpersonal touch condition and the impersonal touch condition. The first condition provided the stroking input to the participant’s (invisible) right forearm with a palm-side of the human hand and the second condition with a thermal probe (see Fig 1a). Either the interpersonal and impersonal touch condition, the participants saw the same experimenter’s hand stroking the rubberhand as visual cues and were asked to report RHI questionnaire and questionnaire on touch pleasantness. After completing the experiment, participants were asked to indicate whether they could discriminate between the different stroking conditions (hand vs. probe) by responding with “yes” or “no.” Additionally, post-experiment questionnaires were administered on-line via Google Forms: the Need-For-Touch-Scale (NFT, [48]), and a questionnaire of CT-optimal touch perception and touch deprivation [44].

### Methods and materials

To provide impersonal touch at similar parameters, we used a custom-designed thermal probe (Fig 1a, TH Systems) kept at $32^{\circ }$C (typical skin-to-skin temperature) and compared to a flat hand as interpersonal touch. The contact surface of the probe was a flat and smooth plate consisting of Oxygen-free copper, measuring 60 mm × 40 mm. The control box of the probe provided fine-grained, continuous temperature control of the probe contact surface by means of a water boiling system and thermocouples ($0.1^{\circ }$C resolution).

In each condition, a trained male experimenter, seated in front of the participant, induced a classic RHI paradigm briefly (20s) on the back of the palm and forearm using the fingertips of the right index and middle fingers, and then performed the touch with a flat hand or the probe - participants received a total of 20 times of back-and-forward strokes at a velocity of 3 cm/s on their right forearm. After each condition, participants were asked verbally to rate how each touch made them feel pleasant and how much they experienced the illusion. After rating the touch, there was an interval (60s) for recovery of the CT afferents response which is known to easily become fatigued due to repeated stimulation [14].

### Questionnaires

#### Questionnaire on touch pleasantness

The questionnaire consisted of two questions asking for evaluations of the touch in terms of a positive and a negative connotation: “Did you feel affectionate (such as a warm, stable, or positive feeling) from the touch?” and “Did you feel unpleasant sensations (such as uncomfortableness or dislike) from the touch?” Each question was to be answered on a 7-item Likert-type scale from 1 (strongly disagree) to 7 (strongly agree).

#### Rubber Hand Illusion (RHI) Questionnaire

As we aimed at the ownership aspect of RHI rather than localization, only Statement 3 of the origin version of Rubber Hand Illusion (RHI) Questionnaire was used; “I felt as if the rubber hand was my hand.” Previously, Statement 1 (“It seemed as if I felt the paintbrushes touching my finger where I saw the rubber hand being touched”) or Statement 2 (“It seemed like the touch I felt was caused by the paintbrushes touching the rubber hand”) were found to be closely related to the localization of touch sensation, whereas Statement 3 was solely related to the feeling of ownership itself [49, 50].

#### CT-optimal touch perception and Touch deprivation

We used a short video (about 10 s) that depicted a forearm being stroked with a flat hand with a CT-optimal velocity. Participants were asked to complete a touch perception and touch deprivation questionnaire [44] after watching the video. This was done by rating five statements regarding the pleasantness of the touch (e.g., “How did the video clip make you feel?”) and two statements of touch deprivation (e.g., “Currently, I would prefer to be touched by others.”). In each questionnaire, a mean score was subsequently calculated; higher score in CT-optimal touch perception indicated that the touch observed in the video was perceived as pleasant, whereas higher scores for the touch deprivation indicated that participants felt touch-deprived after having observed the touch. Also, physical living conditions were collected (e.g., “Are you living together with your family?” [Yes;No], “With how many people do you share your house?” [Number of people]).

#### Need for Touch (NFT) scale

To measure the individual differences in preference for touch, we used the 12-item Need For Touch (NFT) Scale [48]. The scale consists of two dimensions; autotelic and instrumental items which measure the preference for the extraction and utilization of information obtained through the haptic system (see [48] for details). This scale is widely used to capture the information of touch behavior when people make purchase decisions. The Autotelic (NFT-A) scale is intended to capture how people like to touch products to have hedonic experience regardless of the purchase goal, whereas the Instrumental (NFT-I) scale is intended to capture how people prefer to touch products in order to acquire information relevant to the purchase decision.

### Analysis

SPSS v. 26 (SPSS Inc., Chicago, IL, USA) was used to analyze the data. Data were checked for normality with Q-Q plots as well as skewness and kurtosis. The latter were within ±2 for all ratings. Also, Q-Q plots demonstrated that scores were approximately normally distributed. Therefore, paired t-tests and regular linear regressions were used to analyze these variables.

The main analysis for the experiment concerned the relationship between the affective ratings and the RHI scores in the two conditions of interpersonal and impersonal touch. Ratings were subjected to a paired t-test to examine the difference of response to the touch forms between affective ratings and the RHI scores. We also conducted a Bayesian analysis on our main effect of interest (i.e., differences between conditions in the pleasantness and the effect of RHI), by focusing on the Bayes factor (BF) as the main output index. The ${\text{BF}}_{01}$ indicates the probability of supporting the null hypothesis (H0, see Introduction) over the alternative hypothesis (H1). For example, a ${\text{BF}}_{01}$ = 6 means that the observed data are 6 times more likely under the H0 compared to the H1 [51]. Additionally, a linear regression was conducted as another main analysis to investigate the relationship between touch deprivation and perceived pleasantness.

In addition to these main analyses, we added exploratory Analyses of Variance (ANOVAs) to examine potential influences of gender and order of touch on the result. Similarly, another exploratory linear regression was used to examine the relationship between affective ratings and NFT scale, and to analyze whether living conditions affect touch deprivation or perceived pleasantness. To characterize the correlations between the different measures, we also performed an overall pair-wise correlation analysis using Pearson correlations, corrected using a standard FDR (false discovery rate) procedure.

## Results

### Differences in touch pleasantness and RHI between interpersonal and impersonal touch conditions

No significant difference in pleasantness was found between interpersonal touch (M=4.47, SD=1.36) and impersonal touch (M=4.36, SD=1.35), t(44)=0.479, *p* = .634, 95% CI[–0.36, 0.58], suggesting that participants experienced similar levels of pleasantness in both touch induction conditions. Similarly, there was no significant difference in the RHI effect between the hand condition (M=3.53, SD=1.83) and the probe condition (M=3.53, SD=1.73), t(44)=0.0, *p* = 1.0, 95% CI[–0.41, 0.41], indicating comparable illusion strength regardless of the touch source. To determine whether our data support the null hypothesis of no difference between the effects of pleasantness and the RHI effect, we also performed Bayesian statistics on our main effect of interest, specifically examining the differences between touch conditions in the perception of touch pleasantness and the effect of RHI. The ${\text{BF}}_{01}$ represents the likelihood of the null hypothesis compared to the alternative hypothesis. Evidence supporting the alternative hypothesis is indicated by a ${\text{BF}}_{01}$ < 1, whereas evidence supporting the null hypothesis is indicated by a ${\text{BF}}_{01}$ > 1 [52]. The analysis yielded a ${\text{BF}}_{01}$ of 7.674 and 8.587 in favor of the null hypothesis, indicating no significant difference in pleasantness and the RHI effect between interpersonal and impersonal touch conditions. This result suggests that the observed data are 7.674 and 8.587 times more likely under the null hypothesis compared to the alternative hypothesis.

**Fig 2:**
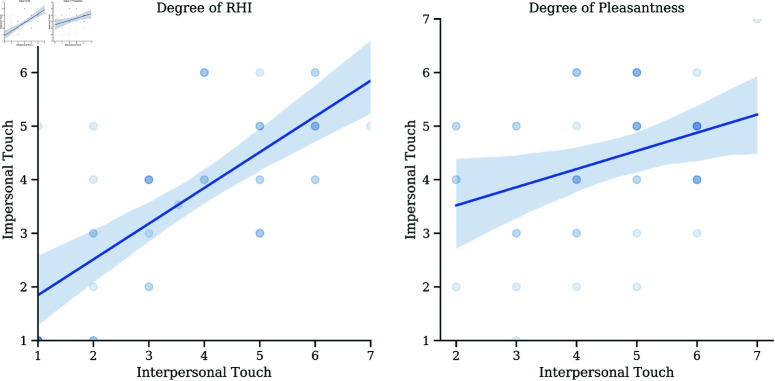
Correlation between interpersonal and impersonal touch. (a) Degree of RHI between the touch conditions, Pearson *r* = .707, *p* < .001, 95% CI[–0.41, 0.41]. (b) Degree of pleasantness between the touch conditions, Pearson *r* = .341, *p* = .022, 95% CI[–0.36, 0.58].

Pearson correlation analysis also indicated a significant association between touch conditions in both pleasantness (Pearson *r* = .341, *p* = .022) and RHI (Pearson *r* = .707, *p* < .001)(Fig 2). Our additional exploratory analyses of potential sex differences and of order of the touch conditions on the pleasantness and RHI measures found no effects on affective ratings: RHI order: F(1,41)=0.806, *p* = .424; RHI sex: F(1,41)=0.768, *p* = .435, pleasantness order: F(1,41)=0.253, *p* = .618, pleasantness sex: F(1,41)=1.733, *p* = .195.

### Prediction of perceived pleasantness from touch deprivation

To examine whether touch deprivation had any influence on the result, we followed [44] and regarded participants rated higher than average score (>4) on the deprivation scale as touch deprived, which corresponded to 68.9% of the participants (average score on the touch deprivation questionnaire *td* = 4.79 (SD=1.19)). A simple linear regression showed that perceived pleasantness and touch deprivation were related: *t* = 4.59, *p* < .001, showing that touch-deprived participants tended to experience the affective touch as more pleasant. Further exploratory analyses whether participants’ living conditions may have had effects on touch deprivation or the experimental measures we conducted a multiple linear regression (see Table 1). Neither perceived pleasantness (F(3,41)=0.115, *p* = .951, *R*^2^ = .008) or touch deprivation (F(3,41)=1.032, *p* = .388, *R*^2^ = .070) was significantly associated with living conditions.

**Table 1:** Mean perceived pleasantness and touch deprivation scores and multiple linear regression model (95% bias-corrected and accelerated CI reported in parentheses) for living conditions. Reference group: Live alone and in relationship

		Observed pleasantness					Touch deprivation				
**Living condition**	**N**	**Mean(SD)**	**B**	**Std. error**	** β **	**p**	**Mean(SD)**	**B**	**Std. error**	** β **	**p**
**Live alone**	**31**	**4.72(1.19)**	**-**				**4.87(1.22)**	**-**		
(Constant)	19	4.77(1.1)	4.77(4.22, 5.31)	0.27	–	.000	5.11(1.02)	5.11(4.56, 5.66)	0.27	–	.000
**No relationship**	12	4.63(1.37)	–0.14(–1.01, 0.74)	0.43	–0.05	.757	4.5(1.46)	–0.61(–1.5, 0.28)	0.44	–0.23	.175
**Living with others**	**14**	**4.71(1.08)**	**-**				**4.61(1.13)**	**-**		
**In relationship**	8	4.85(1.02)	0.08(–.92, 1.08)	0.50	0.03	.870	4.38(1.36)	–0.73(–1.74, 0.28)	0.50	–0.24	.152
**No relationship**	6	4.53(1.22)	–0.24(–1.34, 0.88)	0.55	–0.07	.672	4.92(0.74)	–0.19(–1.31, 0.94)	0.56	–0.06	.736

### Effects of touch pleasantness on subjective ownership

An exploratory simple linear regression was conducted to assess whether the illusion effect would be associated with touch pleasantness in either condition. Neither the interpersonal (F(1,43)=3.206, *p* = .080) nor the impersonal touch (F(1,43)=3.316, *p* = .076) yielded significant effects. Furthermore, we examined the relation between the illusion effect and touch pleasantness when excluding non-RHI responders—these were defined as responders whose RHI scores were lower than 3, corresponding to 24% of participants. After exclusion, a simple linear regression was conducted again to assess whether the illusion effect would be associated with touch pleasantness in either condition—this yielded again no significant effects for interpersonal (F(1,27)=1.984, *p* = .170), but significant effects for impersonal touch (F(1,30)=7.600, *p* = .010).

### Prediction of touch pleasantness from touch preference

We also looked at potential links between the NFT scale and both experimental measures as an exploratory analysis using linear regression: for the interpersonal condition, neither the NFT-I (F(1,43)=3.440, *p* = .071) nor the NFT-A (F(1,43)=3.220, *p* = .080) yielded significant effects; this was the same for the impersonal touch condition: NFT-I (F(1,43)=0.108, *p* = .744), NFT-A (F(1,43)=2.101, *p* = .154). The correlation between NFT-I and NFT-A was *r* = .590 (*p* < .001).

### Omnibus exploratory: full correlations between features

**Fig 3:**
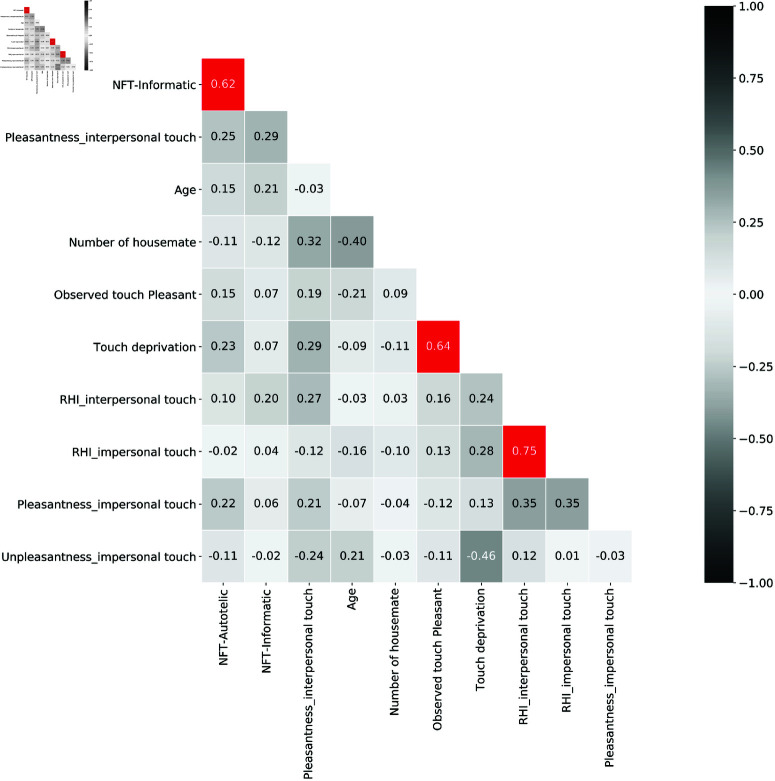
Heatmap of Pearson correlation coefficient matrix. The correlation coefficient matrix is color coded from white (0) to black ±1. Red boxes indicate significant correlations surviving the target FDR level α=0.05.

As an omnibus exploratory analysis, we finally analyzed the full correlations between touch pleasantness, degree of the RHI, Need For Touch Scale (NFT), CT-optimal touch perception and Touch deprivation(see Fig 3). Using a False Discovery Rate (FDR) threshold of *p* < 0.05, we found surviving, significant correlations for RHI ratings of the two conditions of the experiment, for ratings of the two NFT scales, as well as of touch pleasantness and touch deprivation.

## Discussion

The main objective of the present study was to investigate for the first time the relationship between CT-optimal interpersonal touch and impersonal touch with the body-ownership illusion. To achieve this goal, we conducted affective touch with two touch vehicles, a flat hand and the thermal probe, under the RHI paradigm. The impersonal manipulation successfully altered the interpersonal touch in the aspect of pleasantness and embodiment illusion. The results confirmed our hypothesis that impersonal touch with CT-optimal factors (that is, slow gentle touch at skin temperatures) conveys pleasantness and body-ownership illusion similar to interpersonal touch.

Overall, we found that impersonal touch with CT-optimal components under the RHI paradigm was able to provide pleasantness and ownership illusion compared to interpersonal affective touch. During the experiment, the majority of participants (33 out of 45, 73.3%) failed to discriminate between the flat hand and the probe stroking - at the same time, the impersonal touch condition significantly decreased in pleasantness when the participants were aware that they were not stroked by the experimenter’s hand. Specifically, participants who did not perceive a difference rated the probe stroking as more pleasant (M=4.61, SD=1.30) compared to those who were aware of the difference (M=3.67, SD=1.30), t(16.37)=2.14, *p* = .045. This finding suggests that top-down processing influences modulate the perceived touch pleasantness, with awareness of the impersonal nature of the touch reducing subjective pleasantness. Pleasantness ratings for both types of touches overall fell in the middle of the positive side of the response scale, at an overall neutral level of subjective experience of the illusion. This may suggest that even the minimal degree of the ownership illusion may be able to induce the interpersonal affective touch perception regardless of the touch vehicle. When the same hand stroking was performed on a fake forearm, while affective touch on the real forearm was performed with either a hand or the probe, we found a similar sense of perceived pleasantness, but only when the fake arm was embodied at least partially. These findings are consistent with previous research on a role of embodiment in the affective touch (e.g., [35]).

Contrary to our prediction, the level of touch deprivation failed to yield links to perceived pleasantness in both touch conditions - we did, however, replicate previous results [44] showing that the level of touch deprivation correlates to the degree of *o*bserved touch pleasantness. Indeed, people react differently to touch depending on their social context (e.g., [53, 54]) and their individual differences [24]. This is consistent with previous studies that have shown a relationship between early tactile experiences [25, 55] and perceived tactile pleasantness in adulthood. Specifically, studies have indicated that early tactile interactions can have lasting effects on how touch is perceived later in life. In our experiment, the setting of being touched by an (only briefly-familiarized) experimenter in an experimental environment would perhaps be classified as either invited or uninvited touch depending on personal preference.

Previous studies have critically discussed whether affective components, such as CT-optimal velocity and temperature, have significant effects on the ownership illusion. Our results are in line with a recent study [35] that also failed to find the correlation between pleasantness ratings and the subjective experience of the RHI, and another study [56] that found no significant effect of skin-to-skin contact on RHI. Furthermore, when excluding non-RHI responders, the relation between body-ownership and touch pleasantness was observed only for impersonal touch stimulation, not for interpersonal touch stimulation. As mentioned, since the majority of participants did not discriminate the touch stimulation, which components of affective touch would modulate RHI is still an open question. Taken together, as Spaccasass *et al*. [34] reviewed (see also [35, 57]), it remains to be seen whether affective touch itself modulates the subjective experience of the ownership illusion significantly.

In psychological experiments like the RHI, experimental or experimenter cues—termed demand characteristics—may direct participants’ responses toward expected results [47]. Since we relied solely on subject reports, addressing demand characteristics was crucial. To avoid these effects, our study included participants were naïve to the RHI, providing only procedural information without priori knowledge of expected outcomes (i.e., Participants will experience repeated synchronous touching on their real arm and a fake rubber hand). Also, we focused on subjective reports of body-ownership, without assessing proprioceptive drift or including an asynchronous touch condition. This approach would help to moderate the formation of prior expectations among participants, simplifying the assessment of the illusion experience that was less likely to be confounded by demand characteristics.

Moreover, we also assessed whether individual differences in top-down processing, as measured by NFT scale and CT-optimal touch perception and Touch deprivation questionnaire, would moderate the effects of affective touch on the RHI. We were not able to find direct, strong evidence for this, however, at the omnibus level. In a further exploratory analysis at more fine-grained levels, we found that this also held for individual NFT scales, suggesting that NFT may not be an interoceptive modality relevant to pleasantness for being touched.

## Limitations and future research

First, similar to other studies on touch modulating skin temperature, a small, yet possible confound is that we only controlled the probe and room temperatures, but did not specifically control skin temperatures. The experimenter took care to calibrate skin temperature by grasping the probe before the beginning of the experiment until the probe temperature felt neutral - there could, however, be a difference between the first and last stroking in hand temperature.

We also gathered some feedback from participants as to their touch experience. There was no explicit mentioning of temperature regarding affective evaluation, but we did receive contradicting comments as to why some touch would be perceived as unpleasant (i.e., some said due to high friction, whereas other participants mentioned low friction). We therefore cannot yet fully determine which bottom-up components may directly relate to pleasant touch. As such, future research should try to control skin temperatures during the stroking better.

Second, COVID-19-related restrictions inevitably have implications for social interactions, including touch behaviors. Overall, we observed a positive correlation between the touch deprivation and observed touch pleasantness, which could refer to the fact that participants wanted to receive more intimate touch with the ongoing social distancing period in Korea. Although pointing in compatible directions, the correlations between touch deprivation and the experimental measures did not reach significance. This could perhaps be due to differing individual opinions towards touch (i.e., adult attachment style [8]), resulting in different degrees of wanting to be touched [24] by certain groups of people—the touch giver, a naïve experimenter, for example, was not a person regarded as close, which for some people could have resulted in a less desirable touch experience. Controlling for this, however, was not possible in the present context, and relevant patterns might be uncovered in post COVID-19 times.

Third, the current study was based solely on self-report measures obtained also without an asynchronous control condition during the RHI paradigm. The absence of this control may limit our ability to fully discern whether the reported experiences of body ownership are due to influence-by-demand characteristics. It is possible that participants reported either more or less strong illusion experience or pleasantness than they were actually experiencing. Adding such a control conditions is emphasized in recent reviews (e.g., [58]) and would need to be added in future, modified versions of the experiment.

## Conclusion

Our findings corroborate and extend previous studies on the important role of humanness in the pleasure and disembodiment of touch. We performed affective touch with two touch vehicles—a male experimenter’s flat hand and a thermal probe—under the RHI paradigm. Given the results, impersonal touch with CT-optimal components (3 cm/s touch at skin temperature) provided a pleasantness and subjective illusion experience compared to interpersonal touch under the RHI paradigm. Both touch types fell in the middle of the positive side of the response scale, indicating that even a minimal degree of the ownership illusion can induce interpersonal affective touch perception regardless of the touch vehicle. These findings further point to integration of top-down and bottom-up processing influencing whether a touch stimulation is perceived as pleasant regardless of the touch vehicle.

## Supporting information

S1 File Discussion on the relation between SWASH and RHI. This file contains the analysis result, including figures that illustrate the correlation between subjective SWASH hypnotizability and synchronized RHI ratings for both interpersonal and impersonal touch.
(PDF)
