# Supplementary material for: Can impersonal touch replace interpersonal touch? An investigation using the rubber hand illusion
Source: PLoS One. 2025 May 7;20(5):e0319433. doi: 10.1371/journal.pone.0319433 (PMC12058145; doi:10.1371/journal.pone.0319433)
Supplement: S1 File — This file contains the analysis result, including figures that illustrate the correlation between subjective SWASH hypnotizability and synchronized RHI ratings for both interpersonal and impersonal touch. (PDF) [file pone.0319433.s001.pdf]

Can impersonal touch replace interpersonal touch? An investigation using the rubber hand illusion
Bae and Wallraven

Supplementary Discussion

Relation between SWASH and RHI.

In the original study of RHI by Botvinick and Cohen [1] and following studies (e.g. [2]), about 20% of participants reported reduced or no modulation of body-ownership. Recently, [3] found a substantial relationship between RHI experience and hypnotizability, suggesting that RHI may be driven more by task expectations, i.e., reflecting top-down control instead of multisensory, more bottom-up mechanisms. Since this link, however, is under debate (see [4] who observed no significant relationship between RHI experience and hypnotizability), here we also investigated whether hypnotic suggestibility might modulate susceptibility to the RHI.

The study by Lush et al [3] largely draw on a study involving 353 participants who underwent a classic version of the RHI experiment. Although the current study did not have the statistical power to detect the predicted effect, we nonetheless conducted an exploratory analysis to examine the potential relationship between RHI and hypnotic suggestibility.

We administered the Sussex-Waterloo Scale of Hypnotizability (SWASH [5]) questionnaire for a subset of the participants(n=33, 19 women; mean age = 23.8±2.4, range 19-30, never had experience hypnosis) of our main experiment in the lab. Recorded hypnotizability procedures and questionnaires were delivered via Korean (back translation-verified) items. The SWASH was delivered on screen via PsychoPy v3.0 [6] and participants reported subjective and objective responses on a scale from 0 to 5, and yes or no (see [5] for details). The recorded delivery prevented the possibility of differences in delivery style (e.g., tone and speed) across experimenters. Before completing the questionnaire, participants were instructed to wear headphones and to adjust their volume to a comfortable level using a reference tone. They then listened to the voice actor's pre-recorded introductory statements, hypnotic induction, and a series of imaginative suggestions. Following the delivery of the script, they were asked to write their response on a printed 'Response Booklet' using a pen.

Table 1. Mean response on the subjective and objective scores for each suggestion of SWASH and correlation within SWASH and between SWASH and RHI.

	SWASH		Between SWASH(S/O) and RHI		
	**Subjective score(S)**	**Objective score(O)**	**Within correlation**	**Interpersonal session**	**Impersonal session**
	Mean(SD)	Mean(SD)		correlation	
**1.Hand lowering**	2.76(1.35)	0.61(0.50)	.507	-.132/-.165	.052/.078
**2.Moving hands together**	2.24(1.37)	0.85(0.36)	.264	.044/-.104	.073/.009
**3.Mosquito hallucination**	1.45(1.39)	0.42(0.50)	.475	-.05/.145	.213/.275
**4.Taste hallucination**	1.42(1.36)	0.27(0.45)	.721	-.02/.04	.164/.236
**5.Arm rigidity**	2.33(1.38)	0.55(0.51)	.134	-.048/-.027	.086/.187
**6.Arm immobilisation**	2.09(1.49)	0.42(0.50)	.491	-.265/-.287	-.045/.071
**7.Music hallucination**	0.12(0.42)	0(0.00)	N/A	-.088/-	-.022/-
**8.Negative visual hallucination**	0.45(1.25)	0.12(0.33)	.842	-.282/-.311	-.225/-.285
**9.Amnesia**	1.64(1.39)	0.30(0.47)	.079	-.09/-.195	.155/-.232
**10.Post hypnotic suggestion**	2.36(0.88)	0.64(0.49)	.062	.009/.053	.036/-.152

The correlation between the total subjective scale score and total objective scale score was Pearson r = .528, p=.002. Importantly, however, neither the degree of RHI in interpersonal (Pearson r = -.140, p=.438) nor impersonal (Pearson r = .083, p=.647) touch conditions were correlated with the subjective scale of SWASH. When dividing the participants into two groups based on their subjective SWASH scores, we observed that there were no significant differences in either the interpersonal (t=-.500, p=.620, 95% CI[-1.68, 1.02]) or impersonal touch conditions (t=.153, p=.879, 95% CI[-1.22, 1.42]) on the illusion questions.

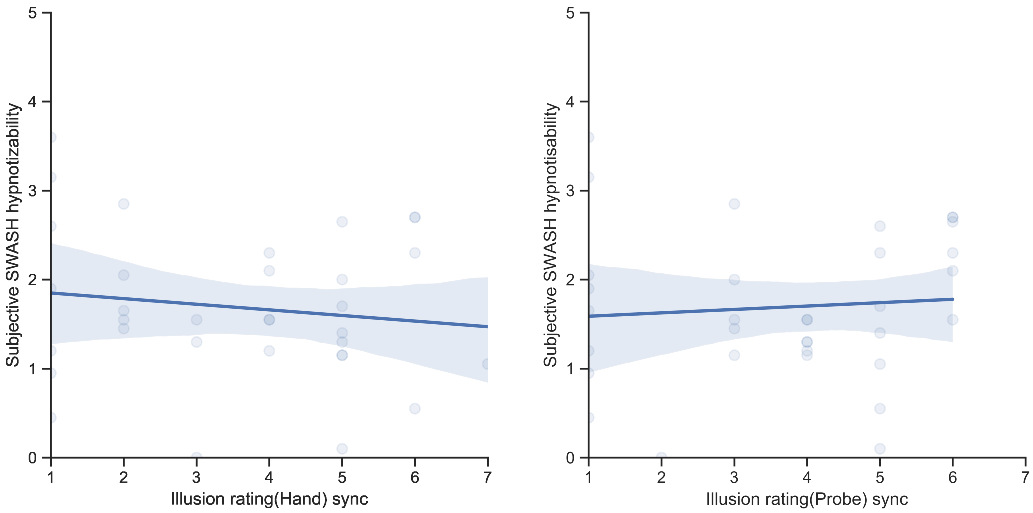

Fig 1. Correlation between subjective SWASH and touch degree of RHI. *Left*, The correlation between total subjective SWASH scale and illusion rating(interpersonal touch condition), Pearson r=-.14, p=.438, 95% CI[1.09, 2.62]. *Right*, The correlation between total subjective SWASH scale and illusion rating(impersonal touch condition), Pearson r=.083, p=.647, 95% CI[1.26, 2.64].

We further conducted a post-hoc power analysis to compare the effect size of the original SWASH study [3] using G*Power 3.1 software [7]. Inputs to the analysis consisted of the correlation coefficient between hypnotizability and degree of RHI in the synchronous stroking condition. Data from [3] (N=353) yielded a small effect size of R^2=0.09, corresponding to 0.999 of power given the large sample size. The current study (N$\bar{3}$3) had effect sizes of R^2=0.02 (0.199 of power) for the interpersonal touch condition and of R^2=0.008 (0.127 of power) for the impersonal touch condition, respectively. Given the very large sample-size of the original study, the failure to replicate the small effect size in the present sample is understandable.

To address individual differences in the experience of the body ownership illusion, we also assessed whether an individual's hypnotizability would moderate the effects of illusion experience on the RHI. Given the potentially lower power of the present study, we were not at first able to replicate the previous findings from Lush et al. [5]. However, a recent review [4] has cast doubt on the purported relationship between hypnotizability and the subjective experience of the illusion. In addition to the lack of an effect for this connection, we also failed to find a strong relationship between SWASH and subjective pleasantness in either touch condition. Together, our findings seem to fit better with the suggestion of Ehrsson et al [4] claiming no specific relationship between hypnotic suggestibility and the RHI.

References

1. Botvinick M, Cohen J. Rubber hands 'feel' touch that eyes see. Nature. 1998;391(6669):756.
2. Riemer M, Trojan J, Beauchamp M, Fuchs X. The rubber hand universe: On the impact of methodological differences in the rubber hand illusion. Neuroscience & Biobehavioral Reviews. 2019;104:268–280.
3. Lush P, Botan V, Scott RB, Seth AK, Ward J, Dienes Z. Trait phenomenological control predicts experience of mirror synaesthesia and the rubber hand illusion. Nature Communications. 2020;11(1):1–10.

4. Ehrsson HH, Fotopoulou A, Radziun D, Longo M, Tsakiris M. No specific relationship between hypnotic suggestibility and the rubber hand illusion. Nature Communications. 2022;13:564.

5. Lush P, Moga G, McLatchie N, Dienes Z. The Sussex-Waterloo Scale of Hypnotizability (SWASH): measuring capacity for altering conscious experience. Neuroscience of Consciousness. 2018;2018(1):niy006.

6. Peirce J, Gray JR, Simpson S, MacAskill M, H"ochenberger R, Sogo H, Kastman E, Lindel"ov JK. PsychoPy2: Experiments in behavior made easy. Behavior Research Methods. 2019;51(1):195–203.

7. Faul F, Erdfelder E, Lang AG, Buchner A. G* Power 3: A flexible statistical power analysis program for the social, behavioral, and biomedical sciences. Behavior Research Methods. 2007;39(2):175–191.
